# *i*Sentenizer-***μ***: Multilingual Sentence Boundary Detection Model

**DOI:** 10.1155/2014/196574

**Published:** 2014-04-15

**Authors:** Derek F. Wong, Lidia S. Chao, Xiaodong Zeng

**Affiliations:** NLPCT Laboratory, Department of Computer and Information Science, University of Macau, Macau

## Abstract

Sentence boundary detection (SBD) system is normally quite sensitive to genres of data that the system is trained on. The genres of data are often referred to the shifts of text topics and new languages domains. Although new detection models can be retrained for different languages or new text genres, previous model has to be thrown away and the creation process has to be restarted from scratch. In this paper, we present a multilingual sentence boundary detection system (*i*Sentenizer-**μ**) for Danish, German, English, Spanish, Dutch, French, Italian, Portuguese, Greek, Finnish, and Swedish languages. The proposed system is able to detect the sentence boundaries of a mixture of different text genres and languages with high accuracy. We employ *i* ^+^Learning algorithm, an incremental tree learning architecture, for constructing the system. * i*Sentenizer-**μ**, under the incremental learning framework, is adaptable to text of different topics and Roman-alphabet languages, by merging new data into existing model to learn the new knowledge incrementally by revision instead of retraining. The system has been extensively evaluated on different languages and text genres and has been compared against two state-of-the-art SBD systems, Punkt and MaxEnt. The experimental results show that the proposed system outperforms the other systems on all datasets.

## 1. Introduction

The task of sentence boundary detection or sentence boundary disambiguation (SBD) is to identify the sentence elements within a text. Many natural language processing (NLP) systems generally take a sentence as an input unit—part of speech (POS) tagging [1], chunking [2], and parsing [3], machine translation (MT) [4], information retrieval (IR) [5], and so forth. The SBD, acting as an initial processing step in most of the NLP applications, seems too simple to get the attention from the researchers. In fact, it is a nontrivial task, since the errors of the SBD system propagate into the subsequent processes when they rely on accurate sentence segmentation, and the overall system performance is negatively affected.

The isolation of sentences involves resolving the use of ambiguous punctuations to determine if the current punctuation is a true delimiter [6]. In English, a period “.,” which is used to signal the end of a sentence, may also be used to denote an abbreviation, acronym, a decimal point, ellipses, and separators in e-mail and World Wide Web addresses. This ambiguity may become serious when the trailing period of an abbreviation or initial also represents the end of a sentence (e.g.,* When I was in Macau S.A.R., I lived on Taipa Island*). Second, the sentence is generally started with capitalized words. If the word following the period is part of proper nouns which is always capitalized, we should not denote the word as the start of the next sentence. All these together make the disambiguation problem more complicated in SBD. On the other hand, the ambiguity of the punctuations varies according to different text genres or specific corpus. In the Wall Street Journal (WSJ), about 42% of the periods denote abbreviations and decimal points while the corresponding percentage for Brown corpus is only 11%. That means if we simply treat every period as the boundary delimiter of a sentence, it is able to correctly detect about 58% of the sentences in the WSJ corpus and about 89% of the Brown corpus. The colon “:,” semicolon “;,” and comma “,” can either be a separator of grammatical subsentences or a delimiter of sentences. About 19% and 14% of colons are being used as the boundary delimiters of sentences in the WSJ and Brown corpus, respectively. This brings another ambiguity of punctuation mark in addition to the period. While semicolon and comma normally are not considered as the end of sentence markers. There are only less than 0.5% of sentences ended with a semicolon or a comma in the WSJ and Brown corpus, and can be negligible. Other possible sentence boundary markers are the exclamation “!” and question “?” marks. They are generally unambiguous in denoting the end of sentences. Hence, the most ambiguous punctuation is the period. In order to implement a reliable SBD model, sophisticated logics and algorithms are required to tackle the problem.

Various algorithms have been employed to achieve the sentence boundary detection in different languages. Recent research works in SBD mainly focus on using machine learning techniques, such as the decision tree [7], the neural network [7], the hidden Markov model [8], the maximum entropy [9], and the conditional random fields [10–12]. They treat the detection task as a classification problem. Although these methods are very successful when applied to an individual language and specific corpus, it remains unclear how well those methods operate if they are applied on a mixture sample of languages and varieties of text genres. Second, the suggested algorithms for the SBD tasks are generally constructed in an offline fashion. Once the model is created, it cannot be altered. Even when the constructed SBD system is adapted to the sample of text which is characteristically different from where it is trained on, it is unable to fine-tune the configuration of the system. This is very important since, in the recent years, there has been a dramatic increase in the collection of online texts for the creation of (multilingual sentence alignment) parallel corpora [13]. The texts are usually of a high variety of sources, genres, domains, and formats over the online content. This poses a special problem for SBD applications. In this paper, we propose a multilingual sentence boundary disambiguation system,* i*Sentenizer-*μ*, as an extension of our previous work [14], based on the incremental learning framework,* i* ^+^Learning principle [15], to deal with the problem we posed. That is different from the existing works reported in the literature.

## 2. Related Work

In the literature, several sentence boundary detection systems have been reported, and basically these systems can be categorized into two types according to the different approaches the systems use. The works of Grefenstette and Tapanainen [6] and Silla et al. [16] are the representative of rule-based approach. The systems encode rules as regular expressions and use set of regular expressions to represent the possible patterns of nonboundary period that mark abbreviations, numbers, and other sequences like email and web addresses. In classification, period in the text is checked and surrounding context is analyzed against the regular expressions. If any regular expression of which is matched, then the period is determined as an abbreviation marker; otherwise, it is considered as a sentence separator. However, rules are never exhaustive, hard to prevent from conflicts, and not robust to the text with domains and genre shifts. The second type of boundary detection systems is based on machine learning approaches. It treats the detection task as a classification problem, using features of the local context of potential boundary markers such as spelling of word, capitalization, length of abbreviation, and part of speech. Compared to manually constructed systems, machine learning models are easier to develop since only annotated training data is required [17]. The representative systems based on this approach are Satz [7] and MxTerminator [9]. The Satz uses either a C4.5 decision tree or a neural network to disambiguate the role of punctuation mark in a sentence, using the prior distributions of word class surrounding the possible end-of-sentence punctuation mark as features. While MxTerminator applies the maximum entropy model to learn the contextual features of ambiguous punctuations by considering the token preceding and following a sentence boundary, together with the heuristic information regarding the abbreviations from the annotated training corpus. Kiss and Strunk [18] propose an unsupervised sentence boundary detection system Punkt that uses collocation information as evidence derived from unannotated corpora for detecting abbreviations, initials, and ordinal numbers. The collocation information and other model parameters are empirically derived from a large development corpus of the Wall Street Journal. Although the system based on machine learning approach is easier to construct, once it is built, the system cannot be changed. In particular, when new domains or text genres are introduced that have different characteristics from its original development data, all the previously learnt knowledge must be discarded. Recently, Wong and Chao [14] propose an online adaptive SBD system to deal with the shifts of text topics on the fly. New data are incrementally learned and are ready to be detected by the revised system. However, the suggested method was tested on two languages only, English and Portuguese. It is unclear how well the performance is if it is applied on a mixed data of wider languages and varieties of text genres. In this research, the proposed system is constructed to detect the boundaries of multilingual text and is extensively tested on eleven different languages and different corpora, including the parallel corpus of Europarl [13].

## 3. Proposed Model

The proposed SBD model is constructed based on* i* ^+^Learning algorithm. The* i* ^+^Learning stands for intelligent, interactive, and dynamic learning architecture, which complements the incremental learning algorithms in terms of performing knowledge revision in multiple dimensions. The algorithm grows an on-going decision tree with respect to either the new incoming instances or attributes in two phases: (1) primary offline construction of decision tree (POFC-DT): a fundamental decision tree construction phase in batch mode that is based on the initial training data and (2) incremental online revision of decision tree (IONR-DT) as incoming of the new instances or feature attributes; existing tree model is revised by incorporating the new knowledge instead of retraining from scratch.

### 3.1. Primary Offline Construction of Decision Tree

This is an ordinary top-down decision tree construction phase that starts from the root node, using a splitting criterion to divide classes as “pure” as possible until a stopping criterion is met. The objective of this phase is to construct an optimal base tree, in order to have a robust foundation for further tree expansion. The binary tree structure is adopted in constructing such base tree. Binary tree has the same representational power as the nonbinary tree, but it is simpler in structure and has no loss of the generated knowledge. This is because the binary decision tree employs a strategy that a complex problem is divided into simpler subproblems, in which it divides an attribute space into two subspaces repeatedly, with the terminal nodes associated with the classes [19].

To build a primitive binary tree, it starts from a root node *d* derived from whichever attribute *a*_*i*_ in an attribute space *A* that minimizes the impurity measure. A binary partition can be denoted by a four-tuple representation (*d*, *T*, *d* ^*L*^, *d* ^*R*^), where *d* is a decision node, *T* is a splitting criterion on *d*, and *d* ^*L*^ and *d* ^*R*^ are the node labels for partitions of the left and right data sets, respectively. Due to a binary tree which is a collection of nested binary partitions, thus it can be represented in the following recursive form:

(1) $$\begin{array}{c} D=\left\{\left(d,T,d^{L},d^{R}\right),D^{L},D^{R}\right\}, \end{array}$$

where *D* ^*L*^ and *D* ^*R*^ denote the left and right subtrees, respectively, which are induced by the partition node *d* [20]. The Kolmogorov-Smirnoff (KS) distance [21, 22] is employed as the measure of impurity at node *d*, which is denoted by *I*_KS_(*d*) and is shown in the equation below:

(2) $$\begin{array}{c} I_{\text{KS}}\left(d\right)=\underset{v\in \text{value}\left(d\right)}{\max}\left|F_{L}\left(v\right)-F_{R}\left(v\right)\right|, \end{array}$$

where *v* denotes either the various values of a nominal attribute *a* with test criterion *a* = *v* or a cut-point of a continuous-valued attribute *a* with test criterion *a* < *v*; *F*_*L*_(*v*) and *F*_*R*_(*v*) are two class-conditional cumulative distribution functions that count the number of instances in the left and right subtrees, respectively, which is partitioned by a value *v* of an attribute *a* at a decision node *d*. KS is a well-known measure for the separability of two distribution functions; it is especially simple and computationally efficient both in the training and classification stages. Hence, a best single test is picked across all attributes by enumerating the possible tests and selecting the one with the greatest KS distance. A decision tree grows by means of successive partitions until a termination criterion is met.

### 3.2. Incremental Online Revision of Decision Tree

IONR-DT phase acts as a central character in the incremental decision tree algorithm. It embraces the faith that whenever a new instance and/or a new attribute is coming, this phase dynamically revises the fundamental tree constructed in POFC-DT phase without sacrificing the final classification accuracy and eventually produces a decision tree as same as possible to those algorithms with all training examples available at the beginning. IONR-DT phase adopts the tree transposition mechanism that in ITI [23] as a basis to grow and revise the base tree. Besides, it preserves the essential statistical information to manage the decision tree. Such style of decision tree differs from the batch mode trees, since it remembers the information of instances regarding the respective possible values as well as the class label, in order to process the transposition without rescanning the entire data set repeatedly.

KS measure is again applied in this phase for evaluating the goodness of a decision node. Once a set of new instances is ready to be incorporated with an existing tree, IONR-DT phase carries out the following several steps. (1) It updates the statistical information on each node that the new instance traversed. (2) It merges the new instance into an existing leaf or grows the tree one level under a leaf. (3) It evaluates the qualification for the test on each node downwards starting from the root node. (4) For any attribute test that is no longer best to be on a node, the pull-up tree transposition process is called recursively to revise the existing decision tree. And (5) a new decision tree is revised and ready to perform the next classification.

### 3.3. Incremental Learning Regarding Attributes (*i* ^+^LRA)

If an instance is available together with an unseen (new) attribute, except the above general steps, an additional procedure for incorporating a new attribute appropriately with an existing decision tree has to be called subsequently. In this algorithm, every new attribute is assigned with a weight of medium importance by default, rather than treated as noise in other learning algorithms [20], although its goodness measurement might be lower on its first occurrence. This is because, for a new domain, when a new attribute is considered to incorporate into the learning process, it should be logically considered as one of the decision nodes, even though the evidence is rare and the attribute might be irrelevant from the statistical point of view.

Further significant, in order to avoid the situation that an attribute has been appended mistakenly,* i* ^+^LRA offers an alternative way to empirically assign one of the four predefined importance grades to an attribute. This characteristic enables* i* ^+^LRA algorithm to be flexible enough to deal with the incremental data appropriately.  Table 1 lists the importance grading schema as well as the respective action being taken during the tree revision process.

### 3.4. Importance Measure (*I*_KS_)

After selecting the importance grade from Table 1 for a new attribute, the crucial step is to determine a preliminary coefficient *W* for its impurity measure (*I*_KS_). This coefficient is vital for a new attribute and is used as a reference index for its importance measure. It is computed only once for each importance grade, in order to enable the new attribute to compete with its comparable attributes in being a decision node. This coefficient is decided as the ratio between the average KS measure of the attributes in the same rank that the new attribute belongs to and the KS measure of the new attribute itself. The following equation illustrates the situation:

(3) $$\begin{array}{c} W=\frac{\text{mean}\left(\sum_{i=1}^{l}I_{\text{KS}}\left(a_{i}\right)\right)}{I_{\text{KS}}\left(a_{\text{new}}\right)}, \end{array}$$

where *a*_new_ is a new incoming attribute; *a*_*i*_ is an attribute of the same rank with *a*_new_; and *l* is the total number of such attributes, which is less than the total number of attributes in the attribute space *A*; that is, *l* ≤ |*A*|. Once such preliminary coefficient *W* for *a*_new_ has been worked out, it could be applied to *a*_new_ by multiplying it to the KS measure of *a*_new_, to enlarge the importance of *a*_new_ according to the given importance grade automatically. This strategy examines the initial importance of *a*_new_ regardless of its KS measure, which can prevent an actually important new attribute being treated as useless due to its newness, and occupies little training data. Thereupon, a normal tree transposition process is carried on as usual to properly fit the new attribute *a*_new_ to a right position.

## 4. Feature Representation

The features we used for constructing the* i*Sentenizer-*μ* are derived from the trigram contexts of training corpus. That includes the words immediately preceding and following the potential boundary punctuation marks: period, exclamation mark, colon, semicolon, question mark, quotation marks, brackets, and dash. Normally, the punctuation marks of the period and exclamation and question marks are only considered as the potential sentence boundaries in related SBD researches [9, 14, 24]. However, there are some cases (depending on different corpora and text genres) where those punctuation marks may also denote a sentence boundary. Second, in order to maximize the adaptability of the system for wider languages and text genres, the associated features are encoded as a binary vector *v* (besides the features representing the length of surrounding words); each component of *v* corresponds to a possible feature value *b*_*i*_ of feature *f*_*i*_ in the feature set. The encoded type of features for constructing the SBD system is therefore independent from specific corpus and alphabet language since the system does not directly rely on the orthographic information. In more details, the information we use consists of the following features.

### 4.1. Character Features

We first consider the capitalization of the initial character of the word, including the immediately preceding word *f*_1_(*w*_*i*−1_) and the following word *f*_2_(*w*_*i*+1_), based on the following feature function:

(4) $$\begin{array}{c} f_{1,2}\left(w_{i}\right)=\{\begin{array}{cc} 1 & c_{1}\in C,\;w_{i}=\left\{c_{1},c_{2},\ldots ,c_{n}\right\}\\ 0 & \text{otherwise}, \end{array} \end{array}$$

where *C* is the collection of capital letters; *c*_*i*_ ∈ *C* × *L* is a character, and *n* is the word length |*w*|. The capitalization of a word gives an important clue to signal the named entities (names of people, places, organizations, artifacts, etc.), abbreviations (e.g., Dr., Mr.), and acronyms (e.g., S.A.R.). Different from previous works of [9, 25], we do not include words prefixes or suffixes in our feature set; consider that affix uses orthographic information and is not independent of any specific language.

### 4.2. Word Features

Under the observation that abbreviations are generally the major source of ambiguities in the determination of sentence boundaries. They are usually short, uppercased, and tightly collocated with internal periods (i.e., acronyms) or a final period (i.e., honorific abbreviations, location name abbreviations, month and measure unit abbreviations, corporate designators, etc.) In addition to the capitalized features, we also care about the upper-/lowercasing of both the neighboring words, *f*_3_(*w*_*i*−1_) and *f*_4_(*w*_*i*+1_), defined as:

(5) $$\begin{array}{c} f_{3,4}\left(w_{i}\right)=\{\begin{array}{cc} 1 & {\forall c}_{i}\in C,\;w_{i}=\left\{c_{1},c_{2},\ldots ,c_{n}\right\}\\ 0 & \text{otherwise}. \end{array} \end{array}$$

Features five and six are considered as the length of previous and next words, that is, *f*_5_(*w*_*i*−1_) and *f*_6_(*w*_*i*+1_), respectively, and are given by

(6) $$\begin{array}{c} f_{5,6}\left(w_{i}\right)=\left|w_{i}\right|. \end{array}$$

### 4.3. Punctuation, Number, and Symbol Features

In previous works [8, 9, 26], features were designed to use the surrounding context in general. However, in order to maximize the capability of detecting the boundaries of sentences in text with different genres and domains, we include also the nonlexical information as features, that concerning the collocation pattern of punctuations, numbers, and symbols. One of the widely used corpora for evaluation in natural language processing tasks is the Wall Street Journal (WSJ) corpus. The WSJ is a typical journalistic news wire corpus. It consists of speech and narratives marked by double (single) quotes. The use of dashes and numeral symbols in the corpus is frequent also. The collocation of those punctuation marks and symbols leads to the ambiguities and makes the boundaries detecting tasks more difficult [27]. Thus, the features that used to capture this information include *f*_7_(*w*_*i*_, *w*_*i*+1_), for detecting the collocation of colon with dash, period, and semicolon:

(7) $$\begin{array}{c} f_{7}\left(w_{i},w_{i+1}\right)=\{\begin{array}{cc} 1 & w_{i}=\text{colon},\;w_{i+1}\in P_{\text{dps}}\\ 0 & \text{otherwise}, \end{array} \end{array}$$

where *P*_dps_ = {dash, period, semi_colon}. The checking of dollar sign *f*_8_(*w*_*i*_) and number *f*_9_(*w*_*i*_) is given by

(8) $$\begin{array}{c} f_{8}\left(w_{i}\right)=\{\begin{array}{cc} 1 & w_{i}="\text{\$}"\\ 0 & \text{otherwise}, \end{array}\\ f_{9}\left(w_{i}\right)=\{\begin{array}{cc} 1 & w_{i}\in N\\ 0 & \text{otherwise}, \end{array} \end{array}$$

where *N* is numeric literals. *f*_10_(*w*_*i*_, *w*_*i*+1_) describes the expression of potential punctuations followed by either dash or left quotation mark:

(9) $$\begin{array}{c} f_{10}\left(w_{i},w_{i+1}\right)=\{\begin{array}{cc} 1 & w_{i}\in P^{*},\;w_{i+1}\in P_{\text{dq}}\\ 0 & \text{otherwise}. \end{array} \end{array}$$

P* is the potential punctuations that signal the boundaries of sentences, and *P*_dq_ = {dash, left_quote}. *f*_11_(*w*_*i*_, *w*_*i*+1_), on the other hand, denotes the expression that excludes left quote which immediately follows the boundary terminal P* and is defined as

(10) $$\begin{array}{c} f_{11}\left(w_{i},w_{i+1}\right)=\{\begin{array}{cc} 1 & w_{i}\in P^{*},\;w_{i+1}\neq P_{q}\\ 0 & \text{otherwise}, \end{array} \end{array}$$

where *P*_*q*_ = left_quote.

### 4.4. Data Representation

In preparation, the corpora used for training and testing the model are transformed by labeling the potential sentence boundaries with **Y** and **N** to indicate the true and false boundaries, respectively, as shown in Box 1.

Instead of feeding the list of sentences into the proposed system directly, the features of potential punctuation are translated as an instance and described in a format of vector 〈*b*_1_, *b*_2_,…, *b*_11_, *l*〉, where *l* is the label (**Y**/**N**) representing if it is the sentence boundary indicator or not.  Box 2 shows the examples of instances construed based on potential trigram contexts.

## 5. Evaluations

### 5.1. Metrics

In order to evaluate the performance of our proposed system, we employ two state-of-the-art sentence boundary detection systems, Punkt (we use the Python version of Punkt provided by the NLTK package (http://www.nltk.org/)) and MaxEnt (it is an implementation of MxTerminator released by Apache OpenNLP package (http://opennlp.apache.org/)), with highest results reported in the literature, for comparison. We treat them as the benchmarks and evaluate* i*Sentenizer-*μ* against these standards. For the performance measure, there are several metrics proposed to evaluate the detection performance of a BSD system. This includes the error rate, *F*-Score, ROC curve, PR curve, and DET curve [28, 29]. Basically, all of those metrics interpret the measure based on the counting of* true* and* false positives* and* true* and* false negatives*, as denoted in the confusion matrix (Table 2).

In this study, instead of measuring the error rate as in [8], we prefer to use the harmonic mean of precision and recall; besides, due to its popularity [18, 27], precision and recall can provide us with better information on what kinds of errors that our system made. Let *T*_*p*_ (true positives) be the number of boundaries in the test data which the system has correctly detected, let *F*_*n*_ (false negatives) be the number of boundaries in the test data which the system has missed, let *F*_*p*_ (false positives) be the number of cases that the system misdetects as the boundaries of sentences, and let *T*_*n*_ (true negatives) be the number of cases that have been determined as nonterminal period. Recall is the proportion of all candidates belonging to the true boundaries that have been determined by the evaluated system:

(11) $$\begin{array}{c} \text{Recall}=\frac{T_{p}}{T_{p}+F_{n}}. \end{array}$$

Precision is the ratio between the number of candidates that have been correctly detected and the number of all candidates that have been determined as the boundary markers:

(12) $$\begin{array}{c} \text{Precision}=\frac{T_{p}}{T_{p}+F_{p}}. \end{array}$$

Hence, the harmonic mean of recall and precision is defined as

(13) $$\begin{array}{c} F\text{-Score}=\frac{2\times \text{recall}\times \text{precision}}{\text{recall}+\text{precision}}. \end{array}$$

### 5.2. Corpora Description

In order to verify if the proposed system is robust and adaptable to varieties of languages and text genres. We have evaluated the* i*Sentenizer-*μ* system on the following corpora.

#### 5.2.1. Brown Corpus and Wall Street Journal (WSJ)

Both are part of the Penn Treebank [30]. They are normally used for evaluating the SBD tasks. The Brown corpus in general English and consists of 15 subcorpora of different genres and topics, ranging from news wires and scientific contents to fiction and transcribed speech. Altogether there are about 500 documents. The WSJ corpus is composed of journalistic news wires and is rich in abbreviations and proper names. There are about 2,500 articles. Documents in these corpora are organized in paragraphs and split into sentences. Sentences are further tokenized into words and annotated with part of speech (POS) information. However, we do not make use of this additional annotation information [27] to support the SBD but extract all necessary data from the splitting sentences.

#### 5.2.2. The Tycho Brahe Corpus

This is a parsed corpus of historical Portuguese [31]. It contains 52 articles, about 20,000 sentences, distributed by the Institute of Mathematics and Statistics of the University of São Paulo. The sentences are manually tagged with POS and syntactic features at the University of Campinas in the lines of the Penn-Helsinki Parse Corpus of Middle English [32]. This corpus represents quite different text genres from others as the articles were written by authors between 14th and 19th centuries. We include this in the evaluation as we want to investigate how well our system performed on the corpus.

#### 5.2.3. Europarl Corpus

This is a parallel corpus of text in 11 languages of the European Union: Danish (da), German (de), Greek (el), English (en), Spanish (es), Finnish (fi), French (fr), Italian (it), Dutch (nl), Portuguese (pt), and Swedish (sv). The Europarl represents the spoken proceedings of the European Parliament over a decade. The corpus is originally created for the research of statistical machine translation systems [13]. This corpus is very large, and its size is over 30 million words (around 1 million sentences) for each of the languages. However, we use only part of them for the evaluation. This corpus provides us with an ideal resource for evaluating the performance of our BSD system on the cross domains (multilingual) data.

The relative data size of each of the used corpora both for training and testing the candidate systems is given in Tables 4 and 5. The test data is held back as unseen samples and is excluded from the training set, which is 10% of its original data.

### 5.3. Features Evaluation

As discussed in Section 4, recognition of abbreviations plays an important role in the SBD task [6]. Majority of the ambiguities are caused by the abbreviations, and it can be greatly reduced if those abbreviations are identified. In order to boost the performance of SBD systems, Mikheev [8] and Kiss and Strunk [18] used a list of abbreviations as additional resources that were extracted either by hand from general-purpose dictionaries or automatically from the corpus. In this study instead of using the enumerated abbreviation list, we specifically constructed an extra feature *f*_12_(*w*_*i*−1_, *w*_*i*_, *w*_*i*+1_) to maximize the performance of our model. This feature is defined as an embedded classifier within the model:

(14) $$\begin{array}{c} f_{12}\left(w_{i-1},w_{i},w_{i+1}\right)=C\left(\langle f_{1}^{\prime },f_{2}^{\prime },\ldots ,f_{k}^{\prime }\rangle \right), \end{array}$$

where *C*(·) is a binary class classifier and *f*′ is a vector feature. It returns 1 if the potential candidate is an abbreviation indicator; otherwise 0 is returned. Theoretically, this extra local feature set *f*′ can be embedded as part of the global feature set *f*, and let the model learn from the training data. In this study, another classifier is constructed under the same learning framework to pregenerate the feature values. To evaluate the effectiveness of the designed features, the following models are created:

- baseline: the model is constructed using the features described in Section 4; that is, *f*_1_ ~ *f*_11_;
- *i*Sentenizer-*μ*: the model includes also the twelfth feature in training; that is, *f*_1_ ~ *f*_12_.

The number of abbreviations in different corpora is given in Table 3. Compared with the Brown and Tycho Brahe corpus, the WSJ corpus has rich abbreviations and proper nouns. This certainly presents a challenge for the SBD models. The two models are trained and evaluated on the WSJ, Brown, and Tycho Brahe corpus, respectively. The evaluation results of the models are shown in Tables 7 and 8. We found that the enhanced model* i*Sentenizer-**μ** gives around 1% improvement in both the recall and precision and *F*-Score, while there are very little and no improvement at all on the Brown and Tycho Brahe corpus. It is not surprising that even the baseline model has achieved a very good performance (*F*-Score: 97.86%). When we look at the classification error of the abbreviations, as demonstrated in Table 9, we found that there are significant error reductions of 94% and 67% on test data of the WSJ and Tycho Brahe corpus, respectively. This proves the effectiveness of the proposed feature set. On the subsequent experiments, proposed model will be trained with these optimized features.

### 5.4. Standard Systems

The results presented in the previous section illustrate that* i*Sentenizer-*μ* is able to achieve a very good performance on corpora from different domains and topics. In this section, we want to compare the performance of it to that of the state-of-the-art SBD systems, that is, Punkt and MxTerminator, which have the highest results reported in the literature.

#### 5.4.1. Punkt

It is an unsupervised SBD model proposed by Kiss and Strunk [18]. They characterize abbreviations as collocations and employ the log-likelihood ratios (log⁡*λ*) to detect abbreviations, initials, and ordinal numbers in terms of global evidence (type-based) and local context (token-based). The system does not rely on orthographic information and heuristic clues.

#### 5.4.2. MxTerminator

This model, proposed by Reynar and Ratnaparkhi [9], is constructed based on maximum entropy. The model learns the contextual features from annotated corpora. It uses features such as the tokens preceding and following a potential sentence boundary, and capitalization information, as well as the affixes of induced abbreviations from the training corpus.

### 5.5. Multilingual and Multidomain Evaluation

In this experiment, we train the* i*Sentenizer-*μ* using the optimized feature set, while the training of Punkt and MxTerminator is performed using the default settings. All the models are trained on the corpora described in Tables 4 and 5. There covers the text in eleven languages and four kinds of text genres (WSJ, Brown, Tycho Brahe, and Europarl). The results achieved on different corpora are given in Tables 6 and 10. The experiments show that* i*Sentenizer-*μ* outperforms the other systems on every corpus. It achieves the best *F*-Scores. Although the precisions of* i*Sentenizer-*μ* on the Tycho Brahe (−0.11%) and Danish Europarl (−1.25%) corpora are slightly lower than that of the MxTerminator, the recalls are much higher, +19.57% and +63.36%, respectively. It is surprising to note that both the MxTerminator and Punkt models obtain unexpected low scores on WSJ corpus. The results are quite different from their reports. The main reason is that the current models do not provide any additional abbreviation list, and it is known that the WSJ corpus is richer with abbreviations and proper nouns.

### 5.6. Cross Corpora Evaluation

In the previous section, the results show that* i*Sentenizer-*μ* is able to achieve a good performance on several corpora. In this second part of the experiment, we want to test if the proposed system is well suited to process different text genres and domains. Three different cross corpora evaluations are carried out; (1) we train the systems on the (English) Brown corpus, and test them on the (Portuguese) Tycho Brahe corpus; (2) the experiment is repeated by training the three SBD systems on the Tycho Brahe corpus and testing them on Brown corpus. The results presented in Tables 11 and 12 clearly show that the overall performance (*F*-Score) of the proposed system is always better than the benchmark SBD systems on both settings; and (3) both the* i*Sentenizer-*μ* and standard systems are trained on the mixture of Europarl corpora (training data) covering 11 languages [13] and evaluated on the test data of those corpora. Once again, the result demonstrates that* i*Sentenizer-*μ* can achieve a very good performance (*F*-Score: 97.47%) in detecting the boundaries of sentences from a high variety of text domains, as shown in Table 13.

### 5.7. Incremental Learning Evaluation

For the third part of the experiment, according to the nature of proposed approach,* i*Sentenizer-*μ* is adaptable to the shifts of text genres and new domains by incrementally learning the new instances that have not been seen in the training data. Considering that it is unable to compare against the benchmark systems, in setting up the experiment, we evaluate the* i*Sententizer- on different corpora. The experiment is conducted as follows: (1) the* i*Sentenizer-*μ* is trained on English data and evaluated on Danish data of the Europarl corpus (*i*Sent_E^∧^D); (2) falsely classified instances are collected and ask* i*Sentenizer-*μ* to learn the new data on the fly and test again on the Danish data (*i*Sent_E+D^∧^D); (3) the process is continued on the German data of the Europarl corpus, namely, testing on German data (*i*Sent_E+D^∧^G) and evaluating again after learning the false data (*i*Sent_E+D+G^∧^G). As shown in Table 14, the results are very promising. They give around 4% to 8% improvement in precision and 2% in *F*-Score. The experiments fully demonstrate that the proposed system is quite robust and works well across different corpora without the need of retraining from scratch.

## 6. Conclusions

In this work, we have presented a multilingual sentence boundary detection system (*i*Sentenizer-*μ*) for Danish, German, English, Spanish, Dutch, French, Italian, Portuguese, Greek, Finnish, and Swedish. Different from the related SBD approaches,* i*Sentenizer-*μ* is proposed based on the incremental tree learning algorithm, which allows the detection system to be adaptable across different corpora by easily incorporating the new data into the model dynamically. The model does not rely on orthographic information. In addition, we further introduce an embedded feature set for detecting abbreviations, one of the most important subtasks of sentence boundary disambiguation. The experimental results reveal that the* i*Sentenizer-*μ* outperforms two state-of-the-art boundary detection systems used for comparison. The* i*Sentenizer-*μ* system (online and offline version) is available at http://nlp2ct.cis.umac.mo/views/utility.html.

## Figures and Tables

**Box 1:**
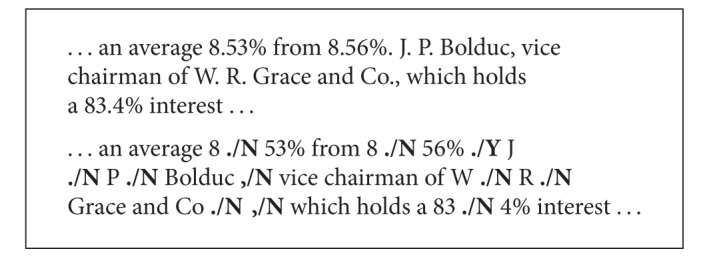
Sentence labeled with boundary tags.

**Box 2:**
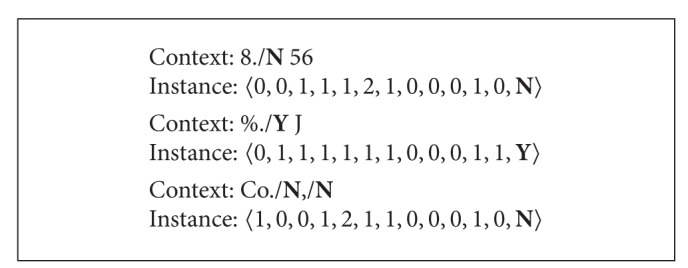
Examples of instances represented as feature vectors.

**Table 1:** Four classes of the importance grades.

Grade	Action in tree revision
High	Attribute *a*_*i*_ should be incorporated into the top several percentages of important decision nodes.

Medium	Attribute *a*_*i*_ can be incorporated into the decision nodes above the average importance.

Low	Attribute *a*_*i*_ may be an irrelevant attribute that is probably appended closer to the leaf node and later on pruned or be considered as a supporting attribute to others.

None	Attribute *a*_*i*_ is treated as noise and can be ignored.

**Table 2:** A confusion matrix for BSD output. “True” denotes positive cases, that is, the sentence boundaries.

	System true	System false
Test true	*T*_*p*_: true Positives	*F*_*n*_: false negatives
Test false	*F*_*p*_: false positives	*T*_*n*_: true negatives

**Table 3:** Number of abbreviations in corpora.

Corpus	Number of abbreviations (train)	Number of abbreviations (test)
WSJ corpus	27,960	3,110
Brown corpus	644	158
Tycho Brahe corpus	382	8

**Table 4:** Size of the Brown, WSJ, and Tycho Brahe corpora.

Corpus	Sentences		Tokens
	Training data	Test data	
WSJ corpus	41,977	4,671	1,153,993
Brown corpus	51,599	5,801	1,155,242
Tycho Brahe corpus	38,000	5,102	953,080

**Table 5:** Information of Europarl corpus.

Language	Sentences		Tokens
	Training data	Test Data	
Danish	30,343	3,375	917,231
German	29,854	3,319	890,176
English	29,774	3,309	949,716
Spanish	33,869	3,765	1,082,826
Dutch	29,604	3,389	688,018
French	29,887	3,321	1,098,724
Italian	27,589	5,067	929,042
Portuguese	28,967	2,777	947,086
Greek	27,687	3,077	888,321
Finnish	29,504	3,309	687,804
Swedish	26,649	2,962	765,795

**Table 6:** Performance of systems on different languages of Europarl corpus.

Corpus	Candidates	Recall	Precision	*F*-Score
Danish	*i*Sentenizer	**98.84%**	92.88%	**95.77%**
	Punkt	97.69%	79.37%	87.59%
	MxTerminator	35.48%	**94.13%**	51.54%

German	*i*Sentenizer	97.61%	**95.77%**	**97.61%**
	Punkt	**97.87%**	87.53%	92.41%
	MxTerminator	81.00%	93.69%	86.89%

English	*i*Sentenizer	**98.98%**	**95.79%**	**97.36%**
	Punkt	97.95%	93.34%	95.59%
	MxTerminator	96.09%	93.97%	95.02%

Spanish	*i*Sentenizer	**99.40%**	**94.21%**	**96.74%**
	Punkt	98.11%	89.80%	93.77%
	MxTerminator	96.67%	90.09%	93.26%

Dutch	*i*Sentenizer	**99.34%**	**96.24%**	**97.77%**
	Punkt	97.79%	92.34%	94.99%
	MxTerminator	91.95%	95.32%	93.61%

French	*i*Sentenizer	**98.82%**	**95.77%**	**97.28%**
	Punkt	97.84%	91.37%	94.49%
	MxTerminator	95.04%	91.88%	93.44%

Italian	*i*Sentenizer	**98.90%**	**95.99%**	**97.42%**
	Punkt	98.25%	93.69%	95.92%
	MxTerminator	94.96%	94.43%	94.70%

Portuguese	*i*Sentenizer	**99.58%**	**96.60%**	**98.07%**
	Punkt	98.50%	95.76%	97.11%
	MxTerminator	94.88%	96.50%	95.68%

Greek	*i*Sentenizer	**97.83%**	**96.44%**	**97.13%**
	Punkt	96.98%	95.36%	96.16%
	MxTerminator	97.24%	93.97%	95.58%

Finnish	*i*Sentenizer	**98.98%**	**95.76%**	**97.34%**
	Punkt	98.33%	92.34%	95.24%
	MxTerminator	92.46%	95.32%	93.87%

Swedish	*i*Sentenizer	95.91%	**94.30%**	**95.10%**
	Punkt	98.94%	89.45%	93.95%
	MxTerminator	**99.49%**	88.33%	93.57%

**Table 7:** Classification results of baseline model.

Corpus	Recall	Precision	*F*-Score
WSJ corpus	0.9757	0.9815	0.9786
Brown corpus	0.9955	0.9995	0.9975
Tycho Brahe corpus	0.9973	0.9983	0.9978

**Table 8:** Classification results of *i*Sentenizer-*μ*.

Corpus	Recall	Precision	*F*-Score
WSJ corpus	0.9843	0.9918	**0.9880 **
Brown corpus	0.9967	0.9995	**0.9981 **
Tycho Brahe corpus	0.9973	0.9983	0.9978

**Table 9:** Abbreviations classification error rate.

Corpus	Baseline	*i*Sentenizer	Reduced error rate
	Errors	Errors	
WSJ corpus	548	34	94% (↓)
Brown corpus	0	0	0%
Tycho Brahe corpus	6	2	67% (↓)

**Table 10:** Results on the Brown, WSJ, and Tycho Brahe corpus.

Corpus	Candidates	Recall	Precision	*F*-Score
WSJ corpus	*i*Sentenizer	**98.48%**	**99.18%**	**98.83%**
	Punkt	93.08%	57.84%	71.34%
	MxTerminator	93.08%	60.24%	73.14%

Brown corpus	*i*Sentenizer	**99.41%**	99.98%	**99.70%**
	Punkt	96.30%	99.95%	98.09%
	MxTerminator	96.30%	99.98%	98.11%

Tycho Brahe corpus	*i*Sentenizer	**99.40%**	99.86%	**99.63%**
	Punkt	79.83%	99.90%	88.74%
	MxTerminator	79.83%	**99.98%**	88.77%

**Table 11:** Trained on the Brown corpus and tested on the Tycho Brahe corpus.

Candidates	Recall	Precision	*F*-Score
*i*Sentenizer	**91.35%**	99.02%	**95.03%**
Punkt	71.80%	99.90%	83.55%
MxTerminator	71.80%	**100.00%**	83.59%

**Table 12:** Trained on the Tycho Brahe corpus and tested on the Brown corpus.

Candidates	Recall	Precision	*F*-Score
*i*Sentenizer	**100.0%**	**95.06%**	**97.47%**
Punkt	95.72%	80.07%	87.20%
MxTerminator	93.54%	93.94%	93.74%

**Table 13:** Results of cross Europarl corpora evaluation.

Candidates	Recall	Precision	*F*-Score
*i*Sentenizer	**100.00%**	94.91%	**97.39%**
Punkt	98.01%	90.11%	93.89%
MxTerminator	92.25%	**95.64%**	93.92%

**Table 14:** Classification results on mixture of Europarl corpora.

Corpus	Recall	Precision	*F*-Score
*i*Sent_E^∧^D	98.15%	86.28%	91.83%
*i*Sent_E + D^∧^D	94.12%	94.59%	94.35%
*i*Sent_E + D^∧^G	96.72%	89.60%	93.02%
*i*Sent_E + D + G^∧^G	96.29%	94.37%	95.32%
